# *Candida krusei* Empyema Thoracis: A Community-Acquired Infection Requiring a High Index of Suspicion

**DOI:** 10.1155/2018/8039803

**Published:** 2018-02-18

**Authors:** Hazim Bukamur, Waseem Ahmed, Yazan Numan, Ibrahim Shahoub, Fuad Zeid

**Affiliations:** Department of Pulmonary Medicine, Joan C. Edwards School of Medicine, Marshall University, Huntington, WV, USA

## Abstract

Empyema thoracis is a serious condition characterized by the accumulation of purulent fluid in the pleural cavity, typically following a pneumonia, subdiaphragmatic abscess, or esophageal rupture. Fungal empyema thoracis is a rare form of this condition with especially high mortality, in which the most frequently isolated fungus is *Candida* spp. This article presents a 74-year-old female with *Candida krusei* pneumonia and a complicated hospital course, initially presenting with nausea, vomiting, and dysphagia. She was initially suspected to have community-acquired pneumonia and was started on azithromycin and ceftriaxone. Worsening respiratory function led to the diagnosis of hydropneumothorax. Pleural fluid and an independent sample of pus and pleural tissue grew *Candida krusei*, giving the diagnosis of fungal empyema. With further respiratory deterioration, the patient was intubated and switched to piperacillin/tazobactam and micafungin. Decortication with extensive pleural peel and removal of foul-smelling pus and food particles within the chest was performed. This further lead to confirmation of esophageal perforation, and she was started on voriconazole and meropenem. After developing septic shock, the patient was managed with phenylephrine and vasopressin. Finally, after improving she was weaned off pressors and extubated, followed by an esophagogastroduodenoscopy (EDG) with pneumatic balloon dilation and WallFlex stent placement. This patient's case demonstrated an example of empyema thoracis, which required a high index of suspicion since the presentation was with a community-acquired infection. *Candida* empyema thoracis may be a complication of operation, gastroesophageal fistula, and spontaneous esophageal rupture. On the other hand, the course of this patient's hospital stay progressed from esophageal perforation to *Candida krusei* pneumonia, empyema, and pneumothorax. Thus, community-acquired fungal empyema should be considered in patients with respiratory symptoms and suspected esophageal perforation; nevertheless, after a diagnosis of fungal empyema, esophageal perforation should also be ruled out in addition to other causes like pneumonia, subphrenic abscess, and hematogenous spread. Improved communication between clinicians and microbiologists can lead to early diagnosis and a reduction in the morbidity and mortality of this condition.

## 1. Introduction

Empyema thoracis is a serious condition characterized by the accumulation of purulent fluid in the pleural cavity, typically following a pneumonia, subdiaphragmatic abscess, or esophageal rupture. Fungal empyema thoracis is a rare form of this condition with especially high mortality, in which the most frequently isolated fungus is *Candida* spp [1]. A high index of suspicion is required to diagnose this condition, as it may present as a community-acquired infection [2].

## 2. Case Presentation

A 74-year-old female with a history of schizophrenia, dementia, COPD, and a CVA presented to the Emergency Department with nausea and vomiting for 3 days, as well as dysphagia for 2 weeks, which all began after drinking hot water. Her vital signs on presentation were BP of 122/77 millimeter of mercury (mmHg), HR of 75 beats per minute (bpm), RR of 16 breaths/minute (br/min), oxygen saturation of 95% on room air, and temperature of 97.7°F. The chest examination revealed decreased air entry and diffuse rhonchi bilaterally. The remainder of physical examination was unremarkable. Her labs showed WBCs of 10,300/mm^3^, creatinine of 0.77 mg/dl, and a negative urine analysis. The chest X-ray (CXR) revealed left lower lobe consolidation with a left-sided pleural effusion. The abdominal X-ray exhibited dilation of the esophagus. The patient refused a modified barium swallow and a CT of the abdomen. She was started on azithromycin and ceftriaxone for community-acquired pneumonia.

**Figure 1:**
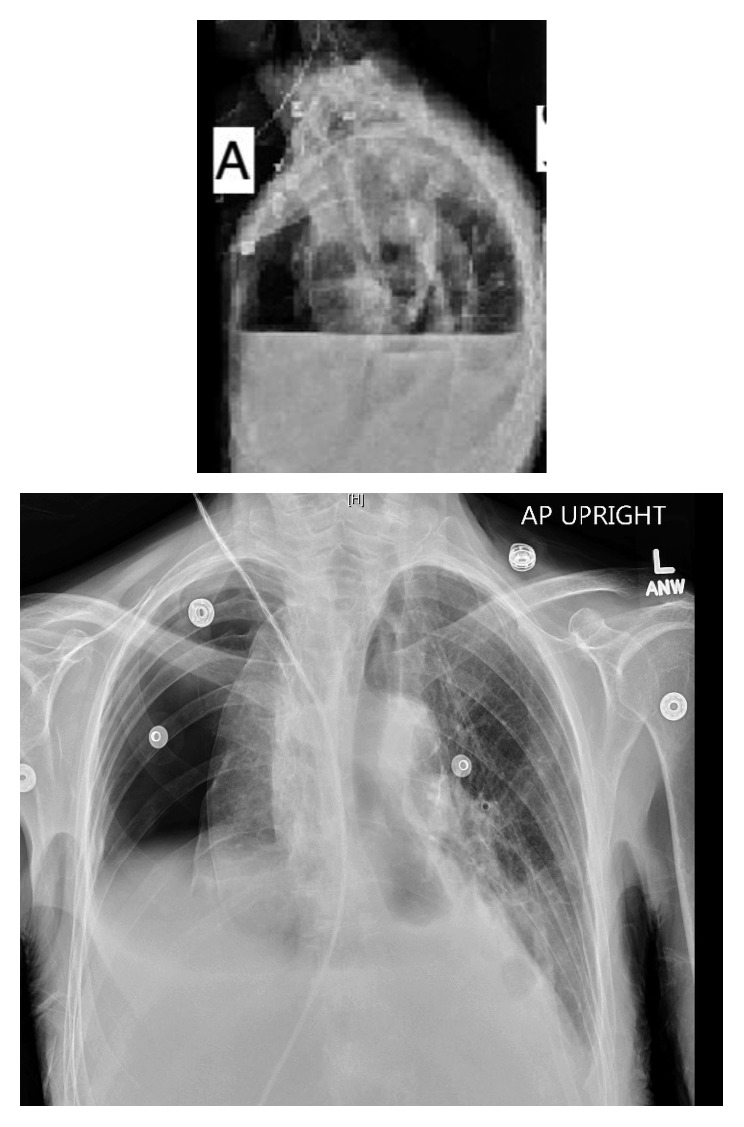
CXR showing a large right-sided hydropneumothorax, and the esophagus was air-filled down to the distal segment.

On the third day of admission, she became short of breath, with vitals of HR 130 bpm, BP 86/43 mmHg, RR 38 br/min, temperature 98.6°F, and oxygen saturation 93% on 3 L/min of oxygen. On examination, the patient was cyanotic, using accessory muscles, taking rapid shallow breaths, with decreased air entry and hyperresonance to percussion on the right side of the chest. The CXR showed a large right-sided hydropneumothorax, and the esophagus was air-filled down to the distal segment (Figure 1). Surgery was consulted, and a chest tube was inserted on the right side, which removed 1.85 liters of pleural fluid. Analysis of the pleural fluid showed WBCs 17424/cmm, RBCs 1728/cmm, neutrophils 95%, lymphocytes 3%, macrocytes 2%, albumin 1.2, amylase 816, glucose < 1, LDH 1559, pH 7.41, and protein 2.7. The pleural fluid grew *Lactobacillus*, and the pleural fluid and sputum culture then grew *Candida krusei*, which was isolated again from a second confirmatory sample of pus, indicating its clinical significance and satisfying the criteria for fungal empyema [2]. Susceptibility testing revealed intermediate sensitivity to 5-flucytosine with minimal inhibitory concentration (MIC) 16.0 mg/L and good sensitivity to itraconazole with MIC of 0.25 mg/L (sensitivity was not done for micafungin). Three sets of blood cultures were negative.

**Figure 2:**
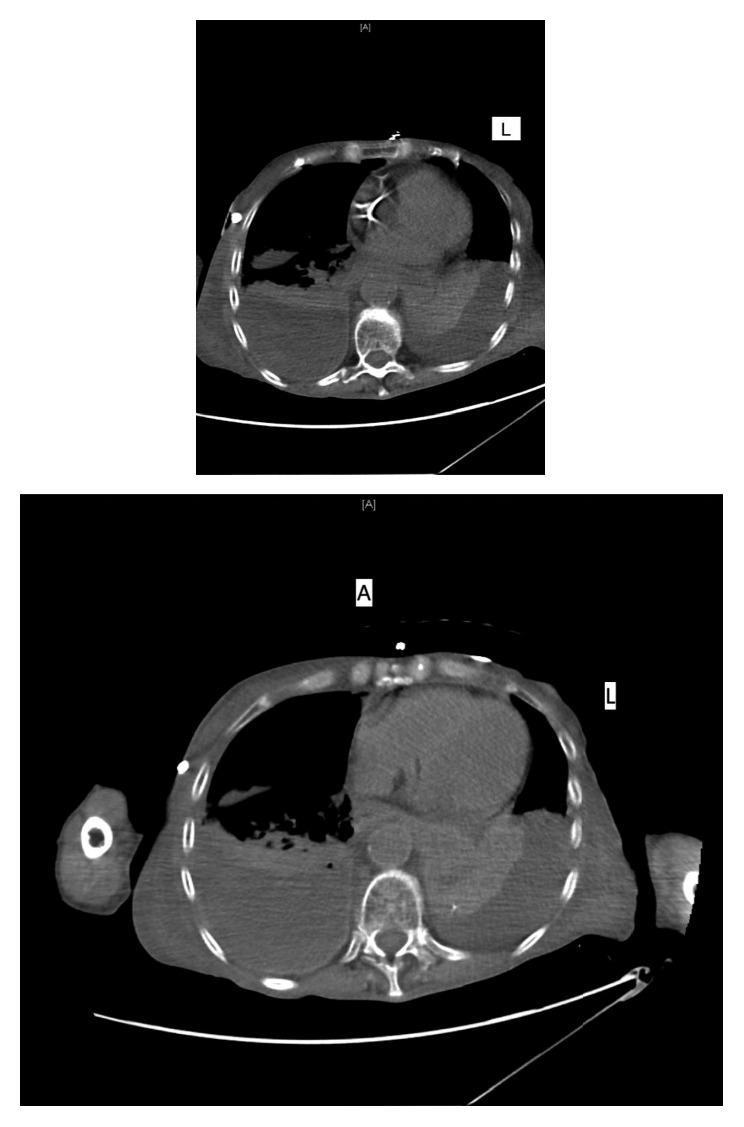
CT of the chest without contrast showing bilateral pleural fluid collections, which were larger and more complex appearing on the right.

**Figure 3:**
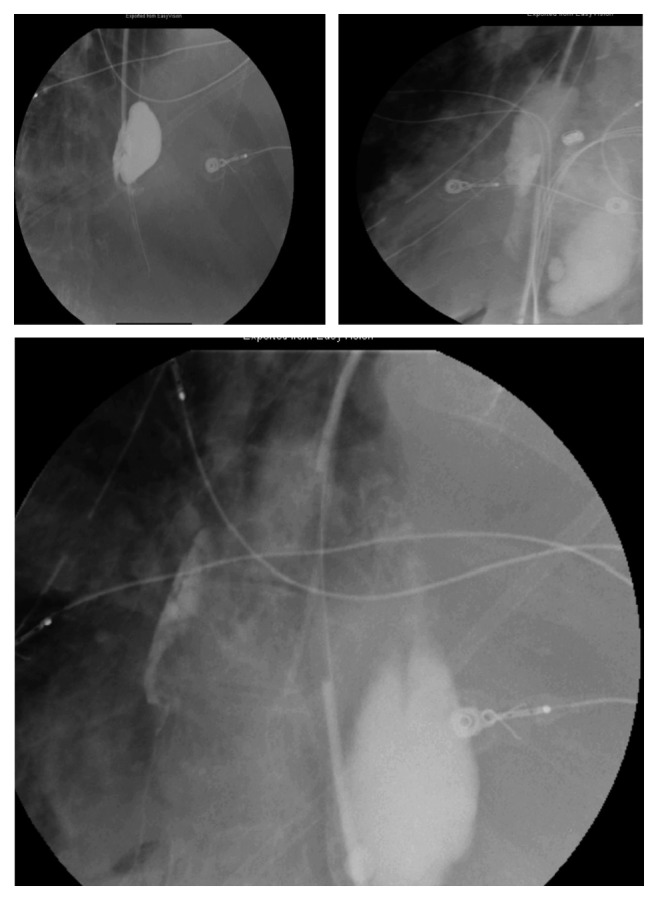
A barium swallow showing contrast extravasation into the right pleural cavity confirming esophageal perforation.

Thereafter, the patient was intubated and transferred to the ICU. The antibiotics were switched to piperacillin/tazobactam and micafungin. CT of the chest without contrast showed bilateral pleural fluid collections, which were larger and more complex appearing on the right (Figure 2). Cardiothoracic surgery was consulted, after which right decortication was performed, with extensive pleural peel and removal of foul-smelling pus and food particles within the chest. Pleural tissue culture grew *C. Krusei*. To further confirm the diagnosis of esophageal rupture, a limited Gastrografin swallow was done through the preexisting NG tube while it was pulled back into the distal esophagus. This revealed a leak in the right distal esophagus, after which a barium swallow exposed esophageal perforation with contrast extravasation into the right pleural cavity (Figure 3), that was confirmed by CT scan.

Infectious diseases was consulted, who then switched the medications to voriconazole and meropenem. Subsequently, the patient developed septic shock during the course of her ICU stay and was started on phenylephrine and vasopressin. After two weeks, the patient improved, was weaned off pressors, and was extubated. An EGD was performed, with pneumatic balloon dilation to 20 mm and WallFlex stent placement. Finally, the patient improved and was discharged from the ICU to the general medical floor.

## 3. Discussion

Empyema is, by definition, pus in the pleural space. Pus is a thick, viscous, yellowish fluid formed in infected tissue, composed of WBCs, tissue debris, necrotic tissue, and pathogens [3]. Fungal empyema thoracis is a rare but severe form of invasive candidiasis with high mortality. The most frequently isolated microorganisms in this condition are *Candida* species, whereas other filamentous fungi are rare, as only sporadic cases have been reported. Interestingly, *Candida* empyema thoracis has been reported as a complication of operation, gastropleural fistula, and spontaneous esophageal rupture [4].

In a retrospective analysis of 128 cases of culture-positive pleural effusion, Ishiguro et al. demonstrated that isolation of *Candida* species could be an important clue for empyema due to gastrointestinal perforation [5]. In this case presentation, the patient presented with dysphagia and was diagnosed with esophageal perforation complicated by *C. krusei* pneumonia, empyema, and pneumothorax.

The diagnosis of fungal bronchopulmonary infection is difficult to confirm because fungi isolated from the sputum may represent either pathogens or saprophytes, and invasive diagnostic procedures such as bronchoscopy or lung biopsy are usually necessary [6]. The diagnosis of fungal empyema thoracis requires that the following criteria be met: (1) isolation of fungal species from exudative thoracentesis fluid, (2) significant signs of infection, such as fever (body temperature > 38.3°C) and leukocytosis (WBCs > 10,000/*µ*L), and (3) isolation of the same fungus from pleural fluid and other specimens, including blood, sputum, or surgical wounds, that showed evidence of tissue invasion [2]. The patient in our case presentation was afebrile, with leukocytosis, and *Candida krusei* was isolated from the thoracentesis fluid as well as sputum and tissue cultures from the thoracotomy, thus satisfying two of the criteria mentioned above.

Although the prevalence of *C. krusei* remains low among yeast infections, its intrinsic resistance to fluconazole raises epidemiological and therapeutic concerns. Echinocandins have *in vitro* activity against most *Candida* spp. and are the first-line agents in the treatment of candidemia. Although resistance to echinocandins is still rare, individual cases of *C. krusei* resistance have been reported in recent years, especially with strains that have been under selective pressure [7]. Review of 25,470 isolates of yeasts and 3,216 isolates of filamentous fungi showed voriconazole to have broad-spectrum activity against pathogenic yeasts including intrinsically fluconazole-resistant isolates such as *C. krusei*, dimorphic fungi, opportunistic molds like *Aspergillus* spp., and amphotericin-B-resistant *Aspergillus terreus*, *Fusarium* spp., and *Scedosporium apiospermum*. It displays excellent clinical efficacy in patients with fluconazole-resistant and fluconazole-susceptible *Candida* infections, invasive bone and central nervous system aspergillosis, and various refractory fungal infections [8]. In our case presentation, the patient was first treated empirically with micafungin and then switched to voriconazole after receiving susceptibility testing results.

There are several surgical options available for management of the pleural fluid in patients with empyema, including tube thoracostomy, intrapleural instillation of fibrinolytics, thoracoscopy with breakdown of adhesions or decortication, thoracotomy with breakdown of adhesions and decortication, and open drainage procedures [3]. Some physicians recommend a formal thoracotomy when the patient's condition allows it, since it may lead to an early discharge without tubes and open draining wounds. Pothula and Krellenstein retrospectively studied 90 patients with this hypothesis, which resulted in low morbidity (10%) and very low mortality (8%). The mortalities in the formal thoracotomy group were mostly related to underlying disease and age [9]. The patient in our case presentation had chest tube insertion and bilateral pleural fluid collections shown on CT of the chest, after which right decortication was performed.

## 4. Conclusion

Infections of the pleural cavity remain an important cause of morbidity and mortality despite advances in diagnostic modalities and therapy. We are presenting this case and are confident that it will be a valuable addition to medical literature in raising awareness about community-acquired fungal empyema in patients presenting with respiratory symptoms and suspected esophageal perforation. Additionally, in the presence of a fungal empyema, esophageal rupture should also be considered and ruled out in addition to other causes like pneumonia, subphrenic abscess, and hematogenous spread [10]. In our case, we think that the fungal empyema happened secondary to esophageal rupture as other causes had been excluded with negative blood culture, no subphrenic abscess in CT abdomen, and no recent trauma or thoracic surgical intervention. In order to accurately diagnose fungal empyema, a high level of suspicion is required, as it may be due to a community-acquired infection. Furthermore, there is a need for improved communication between clinicians and microbiologists for early decision making, which will reduce the morbidity and mortality [11]. Finally, empirical systemic antimycotic therapy should always be considered in an ideal multidisciplinary approach to the management of patients with esophageal rupture [10].
